# Suspected doxycycline-induced thrombocytopenia: Two case reports and a review of the literature

**DOI:** 10.1097/MD.0000000000047903

**Published:** 2026-03-06

**Authors:** Fangpeng Ling, Xuemei Chen, Binling Fan, Shiyu Lan, Chao Jin, Cailian Cai, Keshan Wang, Hongyu Liu, Jianning Jiang, Minghua Su

**Affiliations:** a Department of Infectious Diseases, The First Affiliated Hospital of Guangxi Medical University 6 Shuangyong Road, Nanning City, Guangxi Province, China.

**Keywords:** adverse reactions, doxycycline, drug-induced thrombocytopenia

## Abstract

**Rationale:**

Doxycycline is a broad-spectrum antibiotic widely used in clinical practice. The most common adverse reactions are gastrointestinal disturbances, whereas thrombocytopenia caused by doxycycline is rarely reported.

**Patient concerns:**

Two patients developed severe thrombocytopenia shortly after initiating doxycycline therapy, with platelet counts decreasing post-administration precipitously.

**Diagnoses:**

Drug-induced thrombocytopenia was diagnosed based on the close temporal relationship between doxycycline exposure and cytopenia, together with the exclusion of other common etiologies through clinical and laboratory assessment. Platelet recovery commenced only after doxycycline withdrawal.

**Interventions:**

Treatment included immediate discontinuation of doxycycline, corticosteroid therapy, intravenous immunoglobulin, and platelet transfusion. Specific immunomodulatory and thrombopoietic agents, such as avatrombopag and interleukin-11, were also administered.

**Outcomes:**

Both patients exhibited normalized platelet counts following treatment, without major hemorrhagic events, and achieved complete clinical resolution.

**Lessons:**

These cases suggest that clinicians should be aware of this rare but serious adverse effect. Monitoring platelet counts during doxycycline therapy, particularly in complex patients – especially those receiving multiple medications or with underlying coagulopathy, may help with early detection and timely intervention.

## 1. Introduction

Doxycycline is a semisynthetic form of oxytetracycline with that is effective against a broad range of gram-positive and gram-negative bacteria and is routinely used in the management of rickettsial diseases, mycoplasma infections, and chlamydia.^[1]^ Although this drug is widely used and known to cause adverse effects such as gastrointestinal upset, renal dysfunction, and allergic reactions, thrombocytopenia has been documented only on rare occasions. The potential mechanisms of DITP are varied and include immune‑mediated platelet destruction and bone marrow suppression; in the case of doxycycline, available evidence suggests a possible immune‑mediated pathogenesis.^[2]^ In this report, 2 cases of marked thrombocytopenia suspected to be related to doxycycline exposure are described, underscoring the need for heightened awareness of this uncommon risk and the value of monitoring platelet levels to ensure patient safety during therapy.

## 2. Case 1

A 50-year-old male presented with an unexplained persistent fever (37.5°C–38°C) beginning on May 17, 2023, accompanied by chills, fatigue, dizziness, and recurrent headache. No jaundice was observed. On May 22, he was admitted to a local hospital. Physical examination revealed an axillary eschar, and tsutsugamushi disease was diagnosed. The initial laboratory results included a white blood cell (WBC) count of 13.35 × 10^9^/L, red blood cell (RBC) count of 5.2 × 10^12^/L, hemoglobin (Hb) level of 152 g/L, platelet count of 142 × 10^9^/L, alanine aminotransferase (ALT) level of 139 U/L, and aspartate aminotransferase (AST) level of 77 U/L. Doxycycline was started on May 23 at 0.1 g every 12 hours for 7 days, which resolved the fever. A follow-up laboratory test on May 31 revealed the following: WBC, 7.45 × 10^9^/L; RBC, 4.88 × 10^12^/L; Hb, 135 g/L; and platelet count, 3 × 10^9^/L. By June 4, the platelet count remained critically low at 3 × 10^9^/L, with a WBC of 6.52 × 10^9^/L, RBC of 4.35 × 10^12^/L, and Hb of 127 g/L. Diffuse petechiae and ecchymoses were present on both lower limbs. The patient received treatment to increase the platelet count, support hepatic function, maintain fluid balance, provide nutritional supplementation, and manage symptoms. On June 5, he was transferred to our department with the following admission diagnoses: tsutsugamushi disease; suspected drug-induced thrombocytopenia; and hepatic dysfunction.

**Figure 1.**
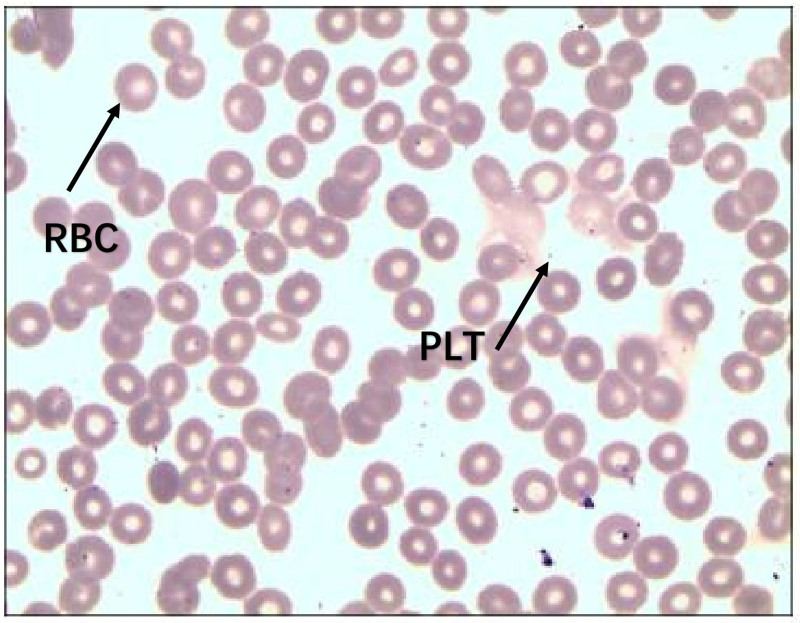
Peripheral PLT morphology (Case 1). Arrow 1: RBCs, exhibiting normal size and hemoglobin content (normocytic, normochromic). Arrow 2: PLTs, showing normal granulation and size. PLTs = platelets, RBCs = red blood cells.

**Figure 2.**
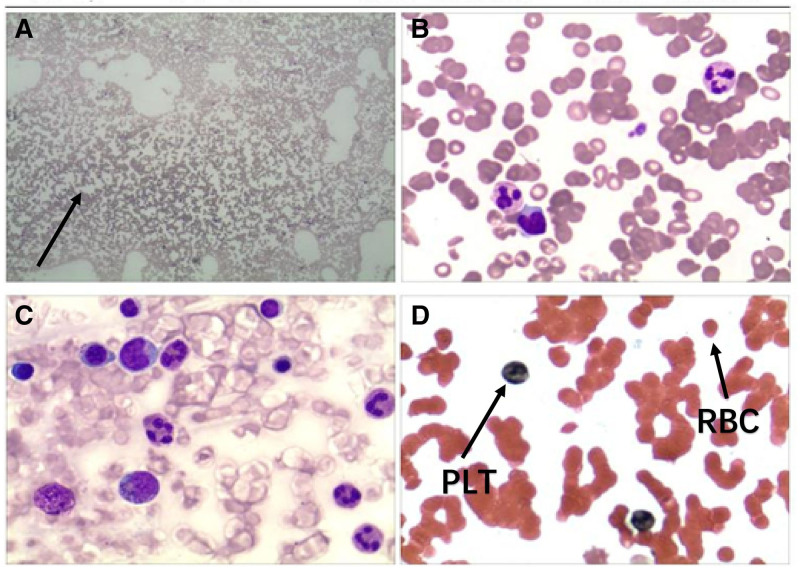
Morphology of bone marrow cells (case 1). (A) Bone marrow smear under low-power microscope, arrows indicate active bone marrow hyperplasia with significantly increased cell density. (B) Bone marrow smear under high-power microscope, showing the morphology of nucleated cells such as granulocytes. (C) Peripheral blood smear, showing lymphocytes and monocytes. (D) Peripheral blood smear, arrow 1 indicates normally shaped red blood cells; arrow 2 indicates platelets, which are significantly reduced in number in the field.

A routine blood test on June 5 revealed the following: RBC count, 4.27 × 10^12^/L; Hb 123 g/L; and platelet count, 2 × 10^9^/L. Microscopic examination (Fig. 1) revealed markedly reduced platelet numbers, with only 0 to 1 platelets per oil-immersion field, and no other notable abnormalities. On June 6, the patient received a 1 U platelet transfusion, along with human interleukin-11 and thrombopoietin receptor agonists. By June 7, the laboratory results revealed an RBC count of 4.29 × 10^12^/L, an Hb concentration of 121 g/L, and a platelet count of 3 × 10^9^/L. An additional 1 U platelet transfusion was given, together with intravenous immunoglobulin (IVIG) (0.5 g/kg) as pulse therapy. Given that the response to transfusions remained limited, avatrombopag was started on June 8, supplemented with interleukin-11 and recombinant human thrombopoietin. On June 9, the patient developed macroscopic hematuria. Bone marrow aspiration and cytologic smear analysis were performed. Etamsylate and carbazochrome sodium sulfonate were administered for symptom relief. Subsequent laboratory tests revealed a platelet count of 3 × 10^9^/L (WBC 11.17 × 10^9^/L, RBC 3.88 × 10^12^/L, Hb 110.9 g/L). On June 10, the platelet count declined to 2 × 10^9^/L, requiring another platelet transfusion. By June 11, the platelet count had decreased further to 1 × 10^9^/L, indicating a continued downward trend. After multidisciplinary consultation, doxycycline-induced thrombocytopenia was strongly suspected. Two additional platelet transfusions were given. Ceftazidime (1.0 g, 10 vials) was administered intravenously once daily for 7 days as infection prophylaxis, together with esomeprazole for gastric protection. High-dose methylprednisolone (MP) (500 mg) and intravenous immunoglobulin (72.5 g/day) were used to counteract antiplatelet antibodies. On June 13, the platelet count increased to 43.5 × 10^9^/L, and methylprednisolone (500 mg) was continued. By June 14, the platelet count had returned to 230 × 10^9^/L. Bone marrow aspiration from 5 sites yielded adequate samples with an overall cellularity of about 30%. Trilineage hematopoiesis was present, with a slightly reduced granulocyte-to-erythrocyte ratio and an increased number of megakaryocytes (16 cells per high-power field in hypercellular regions). No malignant cells were observed. Special stains (Fe/Ag, Periodic acid–Schiff, Giemsa, and Masson) were negative. Pulse therapy with 500 mg of methylprednisolone and 72.5 g of intravenous immunoglobulin was continued. The laboratory results on June 15 revealed the following: RBC count, 3.28 × 10^12^/L; Hb, 92.10 g/L; and platelet count, 400.40 × 10^9^/L. Bone marrow morphology and histochemistry (Fig. 2) revealed hypercellularity, with granulocytes accounting for 71.5%, mainly mature, displaying toxic granulation, and scattered eosinophilic and basophilic cells. Erythroid precursors accounted for 5%, with minimal anisocytosis among mature RBCs. The percentage of lymphocytes was 12%, and the number of mature monocytes increased. Six megakaryocytes were identified per slide, with occasional platelets visible. No parasites or malignant cells were found. Iron staining revealed extracellular iron without sideroblastic granules; a few nucleated erythroid cells contained intracellular iron deposits but were not counted. The neutrophil alkaline phosphatase score was 166. Methylprednisolone was tapered and discontinued once the platelet count stabilized. Although thrombocytopenia can occur in patients with tsutsugamushi disease, this patient had a normal platelet count on admission, which then decreased sharply after initiation of doxycycline. Bone marrow examination revealed no significant abnormalities. Taken together, these findings indicate that thrombocytopenia was caused by doxycycline administration.

## 3. Case 2

On January 9, 2024, a 75-year-old male presented with an unexplained cough accompanied by chest and abdominal pain and fever exceeding 40°C. He denied dizziness, headache, nausea, or vomiting, and was hospitalized. Blood culture confirmed Salmonella infection, but the patient had a poor response to the initial treatment. On January 21, the patient was transferred to the emergency department of a city hospital with suspected sepsis. Laboratory tests at admission revealed the following: 9.22 × 10^9^/L, Hb 103 g/L, and platelet count 35 × 10^9^/L. The vital signs were as follows: body temperature, 35.5°C; heart rate, 68 bpm; respiratory rate, 15 bpm; and blood pressure, 106/70 mm Hg. As Salmonella had been previously isolated from blood cultures, antimicrobial treatment was initiated on January 21 using imipenem–cilastatin (IMI) (500 mg every 4 hours via an infusion pump) together with moxifloxacin (250 mL once daily intravenously). On January 23, laboratory evaluation revealed a platelet count of 84 × 10^9^/L (WBC 12.72 × 10^9^/L, Hb 115 g/L), leading to platelet transfusion. After transfusion, the count increased to 107 × 10^9^/L. Blood cultures obtained on January 24 confirmed infection with Salmonella group D. Because the clinical response to first- and second-generation cephalosporins, cephamycins, and aminoglycosides was insufficient, moxifloxacin was withdrawn. From January 27 to 30, recurrent fever episodes occurred, with a maximum temperature of 38.7°C. Candida tropicalis was detected in bronchoalveolar lavage fluid, and daily fluconazole therapy was started on January 30. The next day, the patient developed scattered erythematous rashes on the trunk and limbs. The fluconazole treatment was stopped because of suspected drug-related hypersensitivity. By February 1, the platelet count had reached 123 × 10^9^/L (WBC 6.94 × 10^9^/L, Hb 86 g/L). Persistent fever prompted a multidisciplinary assessment on February 2. Computed tomography revealed mild blurring of the abdominal wall. Considering the clinical course and suspicion of peritonitis, doxycycline was added to ongoing imipenem therapy to extend coverage against gram-positive, gram-negative, and atypical pathogens. Oral doxycycline hydrochloride (0.1 g every 12 hours) was started on February 2, and intravenous imipenem–cilastatin was continued. The following day, imipenem–cilastatin was stopped and replaced with piperacillin-tazobactam (TZP) (4.5 g every 8 hours intravenously). On February 4, the platelet count fell sharply to 2 × 10^9^/L, accompanied by extensive petechiae and ecchymoses on the chest and back, and platelet transfusion was needed. On February 5, the platelet count was 1 × 10^9^/L. After specialist consultation, the abrupt decrease in the platelet count raised a strong suspicion of doxycycline-induced thrombocytopenia, and doxycycline was immediately discontinued. High-dose methylprednisolone (500 mg/day) plus intravenous immunoglobulin (1 g/kg/day) was given as pulse therapy for 2 days. Despite treatment, the platelet count decreased to 0 × 10^9^/L on February 6, suggesting the need for another platelet transfusion. The subsequent platelet counts were 2 × 10^9^/L (February 7), 16 × 10^9^/L (February 8), and 26 × 10^9^/L (February 9). The patient was transferred to another local hospital on February 9, where laboratory tests revealed Hb at 89 g/L and platelets at 45 × 10^9^/L; by February 14, these values were 78 g/L and 57 × 10^9^/L, respectively. The patient remained febrile. On February 15, the patient presented to our outpatient clinic and was admitted with a diagnosis of sepsis. The final admission diagnoses were as follows: sepsis due to Salmonella group D; pneumonia; drug-induced thrombocytopenia; and lumbosacral fasciitis.

On February 15, the following laboratory results were obtained: RBC count, 2.6 × 10^12^/L; Hb, 76 g/L; and platelet count, 53 × 10^9^/L. The patient continued to experience recurrent fever. Treatment with imipenem–cilastatin (1 g every 8 hours) was initiated, and the fever was successfully controlled. On February 18, given the patient’s advanced age, immunocompromised state, multiple co-infections, and infection risk associated with prolonged central venous catheterization, voriconazole (0.2 g daily) was added for antifungal prophylaxis. By February 20, the platelet count had increased to 263 × 10^9^/L, although fever persisted. A sputum culture obtained externally identified Klebsiella pneumoniae, and tigecycline (TIG) (50 mg every 12 hours) was added. On February 23, due to persistent fever and a limited response, voriconazole was switched to intravenous administration (0.2 g every 12 hours). By February 26, the platelet count had risen to 472 × 10^9^/L. The patient became afebrile on February 27, with improvement in inflammatory markers. The antibiotic regimen was stepped down to piperacillin-tazobactam (4.5 g every 8 hours) after the discontinuation of imipenem–cilastatin, tigecycline, and voriconazole. On March 4, the platelet count was 473 × 10^9^/L, and no fever recurrence occurred. The patient was discharged on March 4, 2024.

## 4. Results and discussion

**Figure 3.**
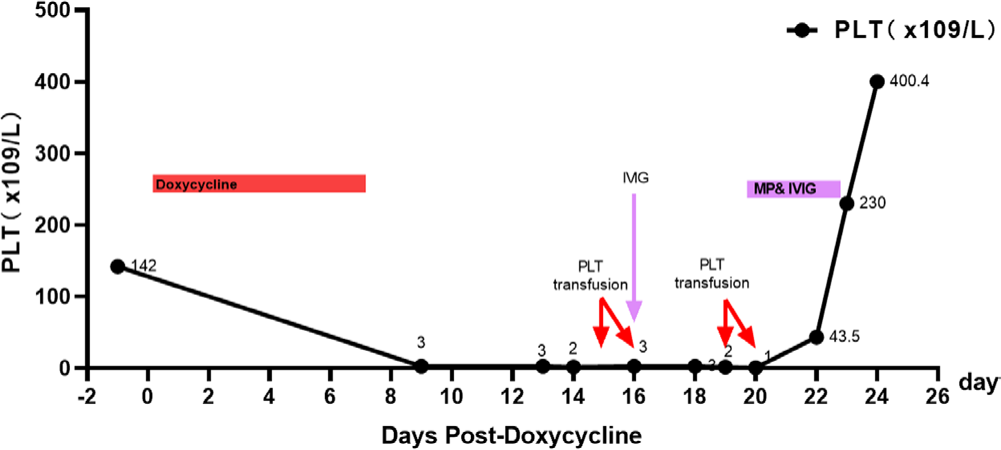
Platelet counts and major interventions during hospitalization (case 1). Red arrow 1, 2, 4, 5: Represents PLT transfusion; purple arrow 3: represents IVIG administration. Shaded areas: the shaded regions indicate periods of treatment; red shaded area: doxycycline administration period. Purple shaded area: MP&IVIG administration period. Data Points: The data points (dots along the line) represent the PLT count values measured at specific days post-doxycycline. The line graph connects these points to show the sequence and trend of platelet counts over time. IVIG = intravenous immunoglobulin, MP&IVIG = methylprednisolone combined with intravenous immunoglobulin, PLT = platelet.

**Figure 4.**
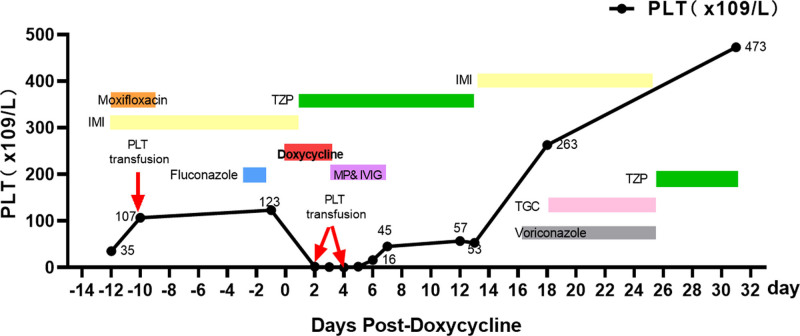
Platelet counts and major interventions during hospitalization (case 2). Red arrow 1, 2, 3: represents PLT transfusion. Shaded areas: the shaded regions indicate the duration of administration for specific medications. Orange shaded area: moxifloxacin administration period. Red shaded area: doxycycline administration period. Purple shaded area: MP&IVIG administration period. Green shaded area: TZP administration period. Yellow shaded area: IMI administration period. Pink shaded area: TGC: administration period. Blue shaded area: fluconazole administration period. Gray shaded area: voriconazole: administration period. Data points: the data points (dots along the line) represent the PLT count values measured at specific days post-doxycycline. The line graph connects these points to show the sequence and trend of platelet counts over time. MP&IVIG = methylprednisolone combined with intravenous immunoglobulin, PLT = platelet, TGC = tigecycline, TZP = piperacillin-tazobactam.

As summarized in Tables 1 and 2 and shown in Figures 3 and 4, both patients developed severe thrombocytopenia (platelet counts falling to single-digit values) shortly after doxycycline initiation, with gradual recovery after its discontinuation. In Case 1, the peripheral smear demonstrated normal platelet morphology (Fig. 1), and bone marrow examination indicated no evidence of malignancy (Fig. 2). The platelet count reached a nadir of 1 × 10^9^/L. After doxycycline was stopped, platelet levels began to rise and returned to the normal range on June 14 following corticosteroid pulse therapy. In Case 2, the initial episode of thrombocytopenia was considered secondary to Salmonella sepsis, as platelet counts improved with the first course of antibiotics. However, an abrupt decrease to a nadir of 0 × 10^9^/L occurred 4 days after doxycycline was initiated for suspected peritonitis. Other simultaneously administered medications – including imipenem, moxifloxacin, and fluconazole – were evaluated. No reduction in platelet levels was observed during therapy with imipenem or moxifloxacin. Although a rash developed with fluconazole, platelet counts remained stable during its use, suggesting an unlikely role for fluconazole. In contrast, the close temporal association between doxycycline exposure and profound thrombocytopenia, along with platelet recovery after its withdrawal (with normalization documented on February 20), strongly suggests a drug-induced cause.

**Table 1:** Platelet count and major interventions (case 1).

Date	Days post-doxycycline	PLT (×10^9^/L)	Major interventions
May 22	−1	142	–
May 23	0	–	Doxycycline initiation
May 29	7	–	Doxycycline withdrawal
May 31	9	3	–
June 4	13	3	–
June 5	14	2	–
June 6	15	–	PLT transfusion
June 7	16	3	PLT transfusion, IVIG
June 9	18	3	–
June 10	19	2	PLT transfusion
June 11	20	1	PLT transfusion, MP, IVIG
June 12	21	–	MP, IVIG
June 13	22	43.5	MP, IVIG
June 14	23	230	MP, IVIG
June 15	24	400.4	–

IVIG = intravenous immunoglobulin, MP = methylprednisolone, PLT = platelet.

**Table 2:** Platelet count and major interventions (case 2).

Date	Days post-doxycycline	PLT (×10^9^/L)	Major interventions
January 21	−12	35	Moxifloxacin, IMI
January 23	−10	107	PLT transfusion
January 24	−9	–	Moxifloxacin withdrawal
January 30	−3	–	Fluconazole
January 31	−2	–	Fluconazole withdrawal
February 1	−1	123	–
February 2	0		Doxycycline
February 3	1		IMI withdrawal, TZP
February 4	2	2	PLT transfusion
February 5	3	1	Doxycycline withdrawal, MP, IVIG
February 6	4	0	PLT transfusion, MP, IVIG
February 7	5	2	MP, IVIG
February 8	6	16	MP, IVIG
February 9	7	45	MP, IVIG
February 14	12	57	–
February 15	13	53	TZP withdrawal, IMI
February 18	16	–	Voriconazole
February 20	18	263	TGC
February 27	25	–	IMI, Voriconazole, TGC withdrawal, TZP
March 4	31	473	TZP withdrawal

IMI = imipenem–cilastatin, IVIG = intravenous immunoglobulin, MP = methylprednisolone, PLT = platelet, TGC = tigecycline, TZP = piperacillin-tazobactam.

## 5. Limitations

Several limitations should be acknowledged. First, definitive serologic confirmation was not obtained, as neither patient underwent testing for drug-dependent antiplatelet antibodies, which is the diagnostic gold standard. Second, in Case 2, bone marrow aspiration was not performed because of the patient’s age and consent issues, so underlying marrow suppression cannot be entirely ruled out. Furthermore, the complex clinical context in Case 2, with multiple concurrent medications and ongoing sepsis-related coagulopathy, complicates the attribution of thrombocytopenia solely to doxycycline. Despite these constraints, the strong temporal association, exclusion of other likely medications, and consistent platelet recovery after discontinuing doxycycline provide compelling clinical support for doxycycline as the probable causative agent.

## 6. Summary

A systematic search of the EMBASE, Cochrane, PubMed, WanFang, and China National Knowledge Infrastructure databases using the keyword “doxycycline adverse reactions” identified numerous documented side effects. Only 1 previously reported case of doxycycline-induced thrombocytopenia, indexed in EMBASE in 2009, was found.^[3]^ Compared with the relatively frequent reporting of other adverse events, the present cases appear to be the first detailed description of this association since 2009, thereby updating the pharmacovigilance profile of doxycycline.

Thrombocytopenia is defined as a platelet count less than 100 × 10^9^/L. Common causes include malignancy, bone marrow suppression, infection, drug induction, hematologic disorders, and autoimmune disease.^[4]^ Drug-induced thrombocytopenia (DITP) is frequently encountered in clinical practice, with more than 300 drugs recognized as potential triggers. Commonly implicated agents include quinine and sulfonamides, and an extensive database (https://www.ouhsc.edu/platelets/ditp.html) catalogs these drugs.^[5–7]^ The proposed mechanisms for DITP include bone marrow suppression, hapten-induced antibodies, quinine-like immune reactions, GPIIb/IIIa inhibition, drug-induced autoantibodies, and heparin-induced thrombocytopenia.^[8]^ In Case 1, bone marrow examination revealed no significant abnormalities, suggesting that marrow suppression was unlikely. Instead, the mechanism of doxycycline-induced thrombocytopenia may involve drug-induced autoantibody formation, as indicated by the persistent thrombocytopenia observed after drug discontinuation.^[9]^

DITP is a diagnosis of exclusion and requires ruling out other possible causes of thrombocytopenia. For example, thrombotic microangiopathy may present with microangiopathic hemolytic anemia and reduced ADAMTS13 activity, whereas sepsis-induced thrombocytopenia is typically accompanied by fever and elevated inflammatory markers.^[5,10]^ The established criteria for implicating a drug include the following: onset coincides with drug initiation and normalization of platelet counts after discontinuation; platelet counts do not decrease after stopping the suspected drug, or the suspected drug is the only drug used before onset; and recurrence of thrombocytopenia with reexposure.^[8,9,11]^ In both cases, reported here, thrombocytopenia developed shortly after doxycycline administration. After other potential medications were excluded and doxycycline, was discontinued, the patient’s platelet counts returned to normal, suggesting a causal relationship. Management of DITP involves immediate withdrawal of the suspected drug and platelet transfusion when clinically needed. Corticosteroids may also be considered.^[10–13]^ In our patients, platelet counts decreased transiently after transfusion. Still, they normalized rapidly following pulse therapy with methylprednisolone and intravenous immunoglobulin, suggesting that a combination of drug cessation, immunosuppression, and supportive transfusion can be effective in severe cases.

These cases highlight doxycycline as a rare cause of clinically significant thrombocytopenia. Clinicians should consider periodic platelet monitoring during therapy, particularly in complex situations, to support early detection and timely intervention.

## Author contributions

**Data curation:** Binling Fan, Shiyu Lan.

**Formal analysis:** Chao Jin.

**Investigation:** Cailian Cai, Keshan Wang.

**Visualization:** Hongyu Liu.

**Writing – original draft:** Fangpeng Ling, Xuemei Chen.

**Writing – review & editing:** Jianning Jiang, Minghua Su.
